# How good are we at implementing evidence to support the management of birth related perineal trauma? A UK wide survey of midwifery practice

**DOI:** 10.1186/1471-2393-12-57

**Published:** 2012-06-25

**Authors:** Debra E Bick, Khaled M Ismail, Sue Macdonald, Peter Thomas, Sue Tohill, Christine Kettle

**Affiliations:** 1 Professor of Evidence Based Midwifery Practice Kings College London, Florence Nightingale School of Nursing and Midwifery, 57 Waterloo Road, London SE1 8WA, UK; 2 School of Clinical & Experimental Medicine, College of Medical & Dental Sciences, University of Birmingham, Birmingham B15 2TT, UK; 3 Education and Research Manager, Royal College of Midwives, 15 Mansfield Street, London WIG 9NH, UK; 4 Centre of Postgraduate Medical Research and Education, School of Health and Social Care, Bournemouth University, R506A Royal London House, Christchurch Road, Bournemouth, Dorset BH1 3LT, UK; 5 Research Manager West Midlands (South) Comprehensive Local Research Network, University Hospitals Coventry and Warwickshire NHS Trust, University Hospital, Clifford Bridge Road, Coventry CV2 2DX, UK; 6 Maternity Centre, Professor of Women’s Health Staffordshire University and University Hospital of North Staffordshire, Newcastle Road, Stoke-on-Trent, Staffordshire ST4 6QG, UK

**Keywords:** Perineal trauma, Episiotomy, Suturing methods, Maternal morbidity, Midwifery practice

## Abstract

**Background:**

The accurate assessment and appropriate repair of birth related perineal trauma require high levels of skill and competency, with evidence based guideline recommendations available to inform UK midwifery practice. Implementation of guideline recommendations could reduce maternal morbidity associated with perineal trauma, which is commonly reported and persistent, with potential to deter women from a future vaginal birth. Despite evidence, limited attention is paid to this important aspect of midwifery practice. We wished to identify how midwives in the UK assessed and repaired perineal trauma and the extent to which practice reflected evidence based guidance. Findings would be used to inform the content of a large intervention study.

**Methods:**

A descriptive cross sectional study was completed. One thousand randomly selected midwives were accessed via the Royal College of Midwives (RCM) and sent a questionnaire. Study inclusion criteria included that the midwives were in clinical practice and undertook perineal assessment and management within their current role. Quantitative and qualitative data were collated. Associations between midwife characteristics and implementation of evidence based recommendations for perineal assessment and management were examined using chi-square tests of association.

**Results:**

405 midwives (40.5%) returned a questionnaire, 338 (83.5%) of whom met inclusion criteria. The majority worked in a consultant led unit (235, 69.5%) and over a third had been qualified for 20 years or longer (129, 38.2%). Compliance with evidence was poor. Few (6%) midwives used evidence based suturing methods to repair all layers of perineal trauma and only 58 (17.3%) performed rectal examination as part of routine perineal trauma assessment. Over half (192, 58.0%) did not suture all second degree tears. Feeling confident to *assess* perineal trauma all of the time was only reported by 116 (34.3%) midwives, with even fewer (73, 21.6%) feeling confident to *perform* perineal repair all of the time. Two thirds of midwives (63.5%) felt confident to perform an episiotomy. Midwives qualified for 20 years or longer and those on more senior clinical grades were most likely to implement evidence based recommendations and feel confident about perineal management.

**Conclusions:**

There are considerable gaps with implementation of evidence to support management of perineal trauma.

## Background

One of the most commonly reported maternal health problems after birth is perineal pain, a symptom highly associated with sustaining perineal trauma during a vaginal birth [1]. It is estimated that approximately 70% of women who have a vaginal birth will sustain perineal trauma which requires suturing [2]. Indeed, for some women, the experience of perineal pain and trauma can impact on their longer-term recovery from the birth [3]. Trauma to the external genitalia can occur spontaneously during a vaginal delivery or as a consequence of a surgical incision (episiotomy) to enlarge the vaginal opening to facilitate birth. Midwives and obstetricians in the UK are expected to document the extent of perineal trauma sustained using a classification system which divides trauma into four types according to the tissues and structures involved [4,5]. Trauma can be classified from a first degree tear which denotes injury to the perineal or vaginal skin only, to a fourth degree tear which is the most severe degree of perineal injury that extends to involve the anal sphincters and anal epithelium [4]. It is essential that clinicians are able to accurately identify and document the extent of trauma to ensure that a woman receives the most appropriate management. Moreover, it is also important that clinicians are able to repair the trauma sustained using suturing methods and materials associated with less short-term perineal pain as recommended by Cochrane systematic reviews [6,7]. Within the UK, these recommendations are incorporated in the NICE Intrapartum Guidelines (4) which inform the care women giving birth in the National Health Service (NHS) in England and Wales should expect to receive.

The Cochrane systematic review of continuous versus interrupted sutures for repair of episiotomy or second degree tears presented data from seven randomised controlled trials and reported that use of a continuous suturing technique for all layers of muscle and skin was associated with less short-term pain compared to interrupted methods. The effect size was greater if the continuous technique was used to repair all layers rather than the skin only [6]. Suturing materials can also affect a woman’s experience of short-term perineal pain. Data from another Cochrane review which included 18 trials presenting data on over 10,000 women found that use of standard synthetic sutures were associated with less perineal pain up to three days post birth and less analgesia use up to ten days postpartum compared with catgut [7]. Rapidly absorbable synthetic sutures were less likely to be associated with the need to remove suture materials. Hence, a rapidly absorbable polyglactin suture (Vicryl Rapide®) is the material of choice within the UK NHS.

The accurate assessment and appropriate repair of perineal trauma require a high level of skill and competency to ensure that the tissues and structures involved are aligned correctly to promote healing and minimise postnatal morbidity. As midwives in the UK are responsible for the majority of normal births, they play an important role in promoting the effective assessment and management of perineal trauma. However, there has been limited attention paid to promoting a high level of skill and competency in this important aspect of maternal health. Midwives may not have been required to undertake further training in perineal repair once qualified. Indeed at the time of conducting this survey, there was no mandatory requirement in the UK to do so. More recently, some NHS hospitals have addressed training provision in order to comply with recommendations of the body which handles negligence claims against the NHS. The Clinical Negligence Scheme for Trusts 2011/2012 Clinical Risk Management Standard 3 requires that a training needs analysis for staff expected to undertake perineal assessment and repair be undertaken [8]. Nevertheless, there is no standard training package in perineal assessment and repair.

Previous studies have highlighted a lack of knowledge with respect to perineal anatomy, classification of trauma and satisfaction with training in perineal repair among midwives and trainee doctors. In one study only 20% of 75 trainee doctors and 48% of 75 midwives questioned reported that they felt their training in perineal repair had been of a high standard [9]. Furthermore, there is anecdotal evidence that the time allocated in the pre-registration midwifery education curriculum in UK universities to teaching the anatomy of the perineum and pelvic floor and training in suturing varies widely, as does the practical experience a student or newly qualified midwife may obtain in the clinical area. There is high quality evidence to support aspects of maternity care globally to prevent or minimise subsequent maternal morbidity, for example reduction in performance of routine episiotomy [10], however there remains a significant ‘evidence to practice’ gap with respect to the assessment and management of the perineum during and immediately following birth [2,11]. Conversely, there has also been limited attention to the impact on clinical skills and competencies of revisions to aspects of perineal management. Barriers to implementing evidence into practice have been identified at many levels including conceptual, instrumental and persuasive use of ‘knowledge’ [12]. As part of the background to the development of a large multi-centre matched pair cluster trial of a quality improvement intervention to promote the accurate assessment and evidence based management of perineal trauma [13], a national survey of UK midwives was undertaken. The study was funded by The Health Foundation and formed work developed for the Perineal Assessment and Repair Longitudinal Study (PEARLS) which is registered in the Current Controlled Trials Registry: ISRCTN28960026.

### Objectives

The main objectives of the survey were to identify how midwives assessed and repaired perineal trauma, including second degree tears and episiotomies, the extent to which their practice ‘matched’ evidence based recommendations, how confident they felt with aspects of perineal management, if they were able to access training to update and maintain their clinical skills and competencies and if national and local protocols to support perineal management were available within their area of practice. The results informed the content of the intervention tested in the Perineal Assessment and Repair Longitudinal (PEARL) Study which is reported separately.

## Methods

A descriptive cross sectional study was undertaken during January to May 2007. The names of 1,000 midwives were randomly selected from the membership database of the Royal College of Midwives, as it was considered that this number would enable a representative sample of midwives to be reflected in final responses. A questionnaire was specifically developed which included a number of closed and open questions, some of which reflected evidence based recommendations for practice. Information on whether the midwife was in practice at the time of the survey and whether they were directly involved in intrapartum care was not available, as these details were not collected by the RCM. Therefore, questions exploring these aspects were included in the questionnaire as both aspects were considered essential criteria for inclusion in the final analyses. The questionnaire included items on confidence in assessing perineal tears, confidence in performing perineal tears and confidence in performing episiotomies rated on a 4 point scale (all of the time, most of the time, some of the time, and never). We were also interested in whether midwives did or did not suture all second degree tears, and the repair technique they used.

Data were entered onto an Access database and analysed using PASW v18. Inclusion criteria included that the midwife was currently in practice and involved in the assessment and repair of perineal trauma. Associations between midwife characteristics and outcomes of interest were explored, using the chi square test for association. Characteristics included number of years qualified as a midwife, the clinical pay scale (or ‘Band’)the midwife was on (‘Band’ refers to nationally agreed pay scales, which reflect the clinical role and responsibilities of midwives, nurses and allied health professionals, a higher Band reflecting a more senior clinical post), the model of maternity care the midwife worked within and the location of care (inner city, urban or rural). These were considered to be characteristics with potential to influence aspects of perineal management. Quotes to illustrate midwives’ responses are included where appropriate.

## Results

### Response rate

A total of 405 (40.5%) midwives returned a questionnaire, 338 (83.5%) of whom met study inclusion criteria.

#### Baseline characteristics of midwives

Of the midwives eligible for inclusion, 306 provided information on their clinical grade, 177 of whom (57.8%) were Band 6, 116 (37.9%) Band 7 and 13 (4.3%) Band 8. A high proportion of midwives had been qualified for over 20 years (129, 38.2%), with 85 (25.1%) qualified for 16 to 20 years, 34 for 11 to 15 years (10.1%), 40 (11.8%) for 6 to 10 years and 50 (14.8%) for 1 to 5 years.

The majority worked in a consultant-led unit (235, 69.5%), with other respondents working in integrated or free-standing midwifery-led units (84, 24.9%). Nineteen (5.6%) midwives were working in independent practice at the time of the survey. In terms of geographic location 171 (50.5%) midwives worked in a unit in an urban location, 84 (24.9%) in an inner city unit, and 83 (24.6%) in a rural area. Due to the small number of Band 8 midwives, the data they provided were combined with data from the Band 7 midwives for purposes of analyses.

#### Perineal trauma management

The midwives were asked what aspects of perineal assessment and repair they undertook and whether they were also required to supervise others undertaking perineal repair. All respondents undertook some aspect of assessment (317, 93.7%) and/or repair (311, 92%), with half reporting that they assessed, repaired and supervised colleagues in this aspect of practice (167/49.4%).

When asked how many perineal repairs they had undertaken in the six month period prior to completing the survey, of the 337 who responded to this question, 104 (30.9%) had not performed any repairs. Of the 233 midwives who had performed repairs, 17 (7.3%) had performed more than 20 repairs, 38 (16.3%) had performed between 10 and 19 repairs, 70 (30.0%) between 5 and 9 repairs and 108 (46.4%) between 1 and 4 repairs.

### Confidence in approaches to perineal management

#### Confidence to assess perineal trauma (n = 338)

Just over a third of the midwives (116, 34.3%) reported that they felt confident to *assess* trauma ‘all’ the time; 186 (55.0%) felt confident ‘most’ of the time and 36 (10.7%) ‘some’ of the time. No midwives reported that they ‘never’ felt confident. There was a statistically significant association with midwifery band and feeling confident, with Band 8 and 7 midwives more likely to report this than Band 6 midwives (p < 0.001, chi square = 21.64, df 2). Sixty-three (48.8%) Band 8 and 7 midwives reported this compared with 43 (24.3%) of Band 6 midwives. There was also a statistically significant association with number of years qualified as a midwife (p < 0.001, chi square = 33.29, df 8); 58 (45.0%) midwives qualified for 20 years or longer were confident ‘all’ of the time compared with 34 (40.0%) midwives qualified for 16 to 20 years, 13 (38.2%) qualified for 11 to 15 years, seven midwives qualified for six to ten years (17.5%) and four (8%) midwives qualified for five years or less. There were no associations with model (p = 0.86) or location of care (p = 0.67).

#### Confidence to perform perineal repair (n = 338)

Only 73 (21.6%) midwives reported that they felt confident to *perform* perineal repair ‘all’ of the time; 183 midwives (54.1%) felt confident ‘most’ of the time and 59 (17.5%) ‘some’ of the time. A small number, 23 (6.8%), reported that they ‘never’ felt confident. When assessed by midwife characteristics, there was a statistically significant association between confidence to perform perineal repair and length of time qualified (p < 0.001, chi square = 57.45, df 12). For those qualified for 20 years or longer 36 (27.9%) reported that they felt confident ‘all’ of the time compared with 23 (27.1%) midwives qualified for 16 to 20 years, 9 (26.5%) of those qualified 11 to 15 years, 3 (7.5%) qualified for 6 to 10 years, and 2 (4.0%) qualified for 1 to 5 years. There was also an association between confidence and band of midwife, with midwives on Bands 8 and 7 more confident to suture than Band 6 midwives (p < 0.001. chi-square =28.24, df 3); 25 (14.1%) Band 6 midwives were confident all of the time compared with 43 (33.3%) midwives in higher bands. Model (p = 0.09) and location of care (p = 0.98) were not associated. Comments added by midwives to this section of the questionnaire illustrated the need to seek support to perform repairs:

"*‘I attempt all repairs that I feel able to perform, however I normally have some assistance at some stage during the repair’ (Hospital Midwife, Band 6)*"

"*‘I feel confident to suture, many colleagues do not and this may be why so many tears are left that should be sutured’ (Hospital Midwife, Band 7)*"

"*‘I take the responsibility seriously, always seek obstetric guidance if needed but have tackled much more difficult traumas than I used to in recent years’ (Hospital Midwife, Band 7)*"

#### Confidence to perform an episiotomy (n = 334)

Two hundred and twelve midwives (63.5%) reported that they were confident ‘all’ of the time to perform an episiotomy, with 73 (21.9%) feeling confident ‘most’ and 38 (11.4%) ‘some’ of the time. Eleven midwives (3.3%) had never performed an episiotomy (10 Band 6 midwives and one Band 7 midwife). When confidence to perform episiotomy was assessed among the 323 midwives who had performed the procedure, there was no association with model (p = .97) or location of care (p = .65). However, there was a significant association between confidence and midwife band. Band 8 and Band 7 midwives were significantly more likely to feel confident than Band 6 midwives (p < 000, chi square =15.21, df 2), for example, 100 (80%) Band 7 and 8 midwives felt confident ‘all’ of the time, compared with 96 (58.9%) Band 6 midwives. Being qualified for 20 years or longer was also significantly associated with feeling confident (p < 0.001, chi square =85.51, df 8). Focussing on being confident ‘all’ of the time, 109 (84.5%) midwives qualified for more than 20 years reported this, compared with 60 (72.3%) midwives qualified for 16 to 20 years, 23 (69.7%) for 11 to 15 years, 11 (32.4%) qualified for six to ten years and eight (21.1%) qualified for five years or less.

The following quotes illustrate the lack of confidence some of the respondents felt:

"*‘with episiotomies, I hardly ever do them, so I have lowered confidence’ (Community Midwife, Band 7)*"

"*‘Hardly ever do an episiotomy, so circumstances would necessitate (ie fetal distress) so less than ideal birth with resulting ‘lowered confidence’ (Community Midwife, Band 7)*"

"*‘I do not like performing episiotomy and always become nervous’ (Hospital Midwife, Band 6)*"

### Suturing materials and suturing techniques

#### Suture materials (n = 331)

Most of the 330 midwives who responded to this question (n = 283, 85.7%) used Vicryl Rapide® for perineal repair. Thirty four (10.3%) reported that they use Vicryl® and nine (2.7%) midwives reported that they used Dexon®. Five midwives (1.5%) did not know what suturing materials were used.

#### Suturing techniques

The midwives were asked which *suturing* techniques they would usually use to repair the vaginal wall, the muscle layer and the perineal skin. The recommended evidence based technique for each layer of perineal repair (continuous non-locking sutures for the vaginal wall and muscle layers, and sub-cutaneous sutures for the perineal skin) were included as possible responses, which enabled the researchers to assess how many midwives were currently using the correct suturing technique to repair all layers of perineal trauma (Table 1).

**Table 1:** Suturing techniques to repair perineal trauma

**Layer of perineal trauma**	
**Vaginal Wall**	**N = 321 (%)**
Continuous locking sutures	190 (59.2)
Continuous non locking sutures*	115 (35.8)
Interrupted sutures	16 (5.0)
Other	0
**Muscle layer**	**N = 323 (%)**
Continuous locking sutures	78 (24.1)
Continuous non locking sutures*	98 (30.3)
Interrupted sutures	147 (45.5)
Other	0
**Perineal skin**	**N = 323 (%)**
Sub-cuticular	156 (48.3)
Sub-cutaneous*	49 (15.2)
Interrupted sutures	102 (31.6)
No sutures inserted	15 (4.6)
Other	1 (0.3)

*the recommended evidence based suturing technique.

There were no statistically significant differences in midwife characteristics examined and use of evidence based suturing techniques for the repair of any layers of perineal trauma, including continuous non-locking sutures for the vaginal wall (p = .47 years qualified, p = .30 location of care, p = .09 band of midwife, p = .99 model of care); continuous non-locking sutures for the muscle layer (p = .40 years qualified, p = .42 location of care, p = .20 band of midwife, p = .40 model of care) or sub-cutaneous suturing of the perineal skin (p = .55 years qualified, p = .16 location of care, p = .50 band of midwife, p = .81 model of care). When responses were examined to elicit how many were using the recommended evidenced based method of repair for all three layers of perineal trauma, only twenty midwives (6%) were using these, 14 of whom had been qualified for 16 years or longer. Ten of the midwives were Band 6.

#### Suturing and non suturing of second degree tears (n = 331)

The midwives were asked if they ever left second degree tears to heal without suturing (‘yes’ or ‘no’). Over half of those who answered this question (192, 58.0%) reported that they did not suture all second degree tears, with no statistically significant differences in how long a midwife had been qualified (p = 0.49), location of practice (p = 0.09) or band of midwife (p = 0.08). When model of care was examined, there was a statistically significant difference with independent midwives more likely to report they would leave second degree tears unsutured (p = 0.009, chi square = 9.52, df 2, with 27 (75.0%) independent midwives reporting this, compared with 44 (67.7%) midwives working in hospital based midwifery led care units and 121 (52.6%) who worked in a consultant led unit. Midwife comments with respect to non-suturing highlighted some of the opposing views with respect to why second degree tears should be repaired, or conversely left to heal with no intervention:

"*‘As a community midwife, I have observed that they often heal better and give less discomfort to clients if left unsutured’ (Community Midwife, Band 7)*"

"*‘Our trust protocol requires that all second degree tears be sutured in practice. If it is thought a small second degree tear would be best left to nature, it might be relabelled as first and left’. (Bank Midwife, Band 6)*"

"*‘The healing process needs some help and support from suturing, especially to realign muscle’. (Hospital Midwife, Band 5)*"

### Rectal examination (n = 335)

When asked if they performed a rectal examination as part of routine assessment of perineal trauma, 142 (42.4%) midwives said they performed this examination ‘all’ of the time, 99 (27.2%) ‘some’ of the time and 44 (13.1%) ‘most’ of the time. Fifty-eight (17.3%) midwives reported that they ‘never’ included this examination as part of the routine assessment of trauma*.* Model of care (p = 0.95), location of practice (p = 0.44) and years qualified as a midwife (p = 0.18) were not associated with this aspect of practice. Performing a rectal examination was associated with band of midwife (p = 0.025, chi square = 9.34, df 3). Focussing on the proportion performing an examination all of the time, 64 (50.4%) Band 8 and 7 midwives reported that they would always perform this examination compared with 64 (35.5%) Band 6 midwives. The majority of the 58 midwives who reported ‘never’ performing rectal examination within routine assessment were in the Band 6 category (40, 74.1%).

Comments from midwives highlighted some confusion as to their role in undertaking this examination:

"*‘I’ve learnt from missed third degree tears from colleagues, so now feel it is better to assess briefly than to miss one’ (Community Midwife, Band 7)*"

"*‘usually the trauma needs suturing which is done by the doctor (if bad enough) who would then perform a rectal examination. I have seen senior midwives suture and not do this’ (Hospital Midwife, Band 6)*"

"*‘I only do this if I think it is a third degree tear, not routinely although this is perhaps a good idea’. (Labour Ward Co-ordinator, Band 8)*"

With respect to performance of a rectal examination *following suturing* of perineal trauma, a higher proportion of the 326 midwives who responded to the question (279, 85.6%) reported they would perform the examination ‘all’ of the time, and 33 (10.1%) ‘most’ of the time, with much smaller proportions reporting that they would ‘never’ perform this (7, 2.1%) or only perform the examination ‘some’ of the time (7, 2.1%). There were no statistically significant differences in band of midwife (p = 0.92), model of care provided (p = 0.68), location (p = 0.79) or number of years qualified (p = 0.10).

### Provision of guidelines, protocols and access to updating of skills and competencies

The majority of midwives (280, 82.8%) reported that they had access to a guideline or protocol for the management and repair of second degree tears, and 267 (79.0%) for the management and repair of episiotomies. When asked who had developed these guidelines or protocols (responses were not separated by type of trauma), 143 (42.3%) midwives reported that they had been developed by the local hospital and 120 (35.5%) that their information was a combination of hospital/RCOG Green Top Guideline. Eleven (3.3%) midwives reported that their organisations only issued the RCOG Green Top Guideline.

Two thirds of the 337 midwives who responded to the question (223, 66.2%) reported that their local maternity service routinely provided structured training for midwives on the management and repair of perineal trauma, and 182 (54.0%) that their service routinely provided *updated* training in these aspects of care. In response to being asked when they had last undertaken any training related to perineal assessment and repair, a higher proportion of the 314 midwives who reported that they had attended training, had received this within the previous two years (162, 51.6%), the remaining 152 (48.4%) midwives undergoing training from three to over ten years previously. The midwives were not asked to provide details of the content of their training. Around two thirds of the 337 midwives (202, 60.0%) reported that their organisation had a standardised proforma for documenting perineal trauma, but only 63 (31.2%) of these 202 midwives reported that this included a diagram to document where the trauma was sustained.

When asked if their competency to undertake assessment and repair of perineal trauma had been formally assessed, less than half of the 333 who answered the question (153, 45.9%) reported that it had with respect to perineal assessment and slightly over half (180, 54.1%) with respect to perineal repair. Of the 304 midwives who answered a question on supervision 93 (30.6%) reported that they felt ‘very’ confident to use the suturing technique recommended in the guidelines/protocols they had access to *without* supervision; 84 midwives (27.6%) did not feel confident and 127 (41.8%) felt ‘fairly’ confident. Only a third of midwives (96, 28.4%) reported that their organisation provided a postnatal information leaflet for women who had sustained perineal trauma.

### Final comments

At the end of the questionnaire, space was left for the midwives who wished to add any further comments. More frequent themes that arose from these were ‘confidence’ and ‘lack of opportunity’ to develop hands-on experience, as the following illustrate:

"*‘I’ve done very little suturing since training, for which supervision was almost impossible to obtain. It seemed easier for the Band 7 midwife or obstetric registrar to do it for you. Therefore competence/confidence remains limited’ (Hospital midwife, Band 6)*"

"*‘It’s difficult to gain experience in perineal repair, as so many doctors during instrumental delivery or there is a complication for example, third degree tear/ragged tear that needs high expertise’ (Hospital Midwife, Band 6)*"

"*‘I participate in labour ward updates frequently and I also inform the coordinator that I require perineal suturing. The opportunity never arises’ (Community Midwife, Band 7)*"

"*‘We are now advised to use continuous suturing – I still prefer the traditional method, interrupted to muscle and to skin, of which I feel more confident’ (Hospital Midwife, Band 6).*"

## Discussion

This is the first national survey to assess the extent to which UK midwives are implementing evidence based management of birth related perineal trauma. Issues have been identified with implications for the education and on-going training of midwives to promote their skills and competencies to ensure the implementation of evidence associated with reduced maternal morbidity. Our inclusion criteria were that midwives were currently in clinical practice and expected to undertake perineal assessment and repair as part of their clinical role. Most midwives reported that they undertook assessment and repair of perineal trauma and around half were also supervising others to repair trauma. Despite this, around a third of midwives had not performed any perineal repairs within the six months prior to completing the questionnaire, and of those who had repaired trauma, most had only performed between one to four repairs. Reasons for such low and infrequent numbers of repairs are unclear, given the large number of UK women who sustain perineal trauma [14], with midwives deemed to be responsible for the suturing of trauma sustained following spontaneous vaginal delivery unless there has been anal sphincter involvement [6]. This finding could explain why so many midwives reported a lack of confidence to assess and repair perineal trauma, with only a third of the midwives feeling confident to *assess* perineal trauma ‘all’ of the time and fewer feeling confident to *repair* trauma, despite access to guidelines and protocols to support practice. Currently, there is no national requirement for midwives or medical staff to perform a certain number of perineal repair procedures per annum to maintain their clinical competency.

In this survey, midwives qualified for longer and those on higher bands (ie the more senior clinical midwives) were more confident in managing perineal trauma. These midwives may have completed their midwifery training and/or been in practice when there was more emphasis on clinical management of the perineum due to use of routine episiotomy. The introduction of restrictive versus routine use of episiotomy in view of the lack of evidence of benefit following routine use [10] resulted in UK episiotomy rates falling from 52% of all vaginal births in England in 1980 to 15% in 2010–2011 [14]. The change in routine practice may have resulted in a gap in the level of experience of ‘hands on’ perineal management midwives can achieve. Moreover, recent changes to the pre-registration midwifery curricula [15] recommend that for entry onto the register as a qualified midwife, the student must be able to initiate emergency measures if required, including episiotomy. However students are no longer required to perform a specific number of episiotomies during their training, with anecdotal evidence that some students have no experience of performing episiotomy on qualifying. Furthermore, it could be postulated that they lack confidence in terms of skills and competencies to support perineal care due to workload pressures and high obstetric intervention rates precluding learning from mentors in practice. Interestingly, a recent postal survey of 1,000 midwives in England, to which 607 (60.7%) replies were received, explored how common a ‘hands off’ approach to perineal management at birth was implemented by midwives [16]. Around half of the midwives (299 (49.3%, 95% CI 45.2-53.3%)) preferred the “hands-off” method, with less experienced midwives more likely to prefer this approach (72% vs. 41.4%, p < 0.001). Furthermore, a higher proportion of midwives in the “hands-off” group would never perform an episiotomy (37.1% vs. 24.4%, p = 0.001) for indications other than fetal distress. Undoubtedly, the above findings suggest an urgent need to consider the subsequent impact that change in practice has on the content of pre registration midwifery training and the development of skills and competencies in perineal management.

The majority of midwives in the current study were using suturing materials for perineal repair associated with reduced maternal morbidity [7]. A Cochrane review found that compared with catgut, standard synthetic sutures were associated with less pain up to three days post delivery (RR 0.83, 95% CI 0.76 to 0.90) and less need for pain relief up to ten days post birth (RR 0.71, 95% CI 0.59 to 0.87), with no evidence of significant differences between groups and perineal pain at three months, or dyspareunia at three or at six to 12 months [7]. This may be an area of evidence based practice relatively easy to disseminate and implement, as use of suturing materials would be an organisation wide decision rather than reliant on an individual clinician.

Any potential benefits to maternal health which may accrue from the use of appropriate suturing material are likely to be dissipated if the individual clinician’s suturing technique is not evidence based. Only a small proportion of the midwives in our survey were using recommended, evidence based suturing techniques to repair all layers of perineal trauma, despite availability of evidence based guidance [4,5]. Furthermore, only a third of the midwives were using continuous non locking sutures to repair the vaginal wall and muscle layers, with fewer using subcutaneous sutures to repair the perineal skin. That only 6% of midwives were using recommended evidence based suturing methods to repair perineal trauma was unexpected. It is acknowledged that there are wide variations in techniques and in materials used for perineal repair between individual practitioners and maternity units [6], with the rationale for the suturing technique chosen appearing to evolve from the way the clinician was taught in the first place, rather than robust clinical evidence. Midwives and student midwives were previously taught to repair perineal trauma using the interrupted method because it was considered easier to learn and may have caused fewer problems in the hands of the inexperienced or novice clinicians [17]. Our data suggest that this continues to be the method most commonly taught, although it has been reported that the continuous technique is simple to perform and could be easily taught to inexperienced clinicians [18]. Use of evidence based suturing techniques was not associated with midwife characteristics examined in the current study, indicating that is a profession wide issue. The failure to promote use of evidence based suturing methods could be viewed as further evidence of a widespread and persistent lack of priority accorded to promote midwifery skills and competencies in perineal management, despite concerns over rising negligence claims for the consequences of poor care [8].

Whilst gaps in practice and evidence implementation are indicative of need for improved basic and on-going training in clinical skills and competencies, conversely some midwives were making decisions about perineal management in the absence of evidence. Non-suturing of second degree tears has persisted in UK midwifery practice, despite current recommendations for practice, based on a systematic review of the evidence, supporting suturing [4]. Two small randomised controlled trials compared suturing versus non-suturing of second-degree tears [19,20] although both lacked statistical power. Lundquist *et al*[19] randomised 80 primiparous women to be sutured or not sutured, collecting data on pain, discomfort and dyspareunia at 2 to 3 days, 8 days and 6 months after birth. Results showed ‘small’ lacerations left unsutured healed as well as ones which were sutured, however the definition and measurement of healing was not clarified, nor was a definition of a ‘small’ laceration provided. Fleming *et al*[20] randomised 74 primparous women who sustained a first or second degree tear to be sutured (n = 33) or not sutured (n = 41). Perineal pain and wound healing were assessed at 1 and 10 days and six weeks postpartum, and postnatal depression assessed at 10 days and six weeks postpartum. No significant differences were found in reported pain or depression, but significantly more women in the sutured group had good wound approximation at six weeks (p < 0.001), further analysis showing that suturing and a shorter labour increased odds of wound approximation.

Current guideline recommendations based on limited evidence that non-suturing is associated with poorer wound healing at 6 weeks post-birth are that all second degree tears should be sutured [4]. Of concern is that the decision not to suture may be being made on the basis of a lack of confidence to assess and repair trauma. The comment from one midwife in the current study that a second degree tear may be deliberately relabelled as a first degree tear to overcome the need to suture, is extremely concerning. The onus to promote accurate recording of a spontaneous tear may not be viewed as important as most midwives reported that they were not required to document the site of perineal trauma sustained using a specific proforma.

Epidemiological and pathophysiological studies generally concur that the two main factors associated with the occurrence of faecal incontinence are instrumental delivery and third/fourth degree tear [21,22]. The incidence of anal sphincter injury has been reported to range from one to eight percent during vaginal birth [23-25], with a more recent systematic review [26] estimating the incidence to be around 11%. Given the increased risk of developing faecal incontinence following anal sphincter injury, there are concerns that more severe perineal trauma may not be correctly identified at the time of birth by midwives or obstetricians [9]. The NICE Intrapartum guideline [4] recommends that systematic assessment of genital trauma following birth should include *‘a rectal examination to assess whether there has been any damage to the external or internal anal sphincter if there is any suspicion that the perineal muscles are damaged’ (p 191).* This recommendation was not routinely implemented by the midwives in the current study, with around a fifth reporting that they would ‘never’ perform a rectal examination as part of routine assessment. Midwife comments illustrate some confusion over their responsibility for completing the examination, with reference to the importance of checking for a third degree tear only if indicated, and a lack of supervisory lead in practice. In contrast, when asked about this aspect of practice *following* suturing, most midwives would perform the examination.

Midwives were able to access training in perineal management, with just over half reporting that their organisation provided updated training in aspects of perineal care, although the content of training, for example in terms of whether this included a ‘hands-on’ element, was not elicited. The midwives’ competencies in aspects of perineal management were, in some cases, formally assessed although again the form of assessment was not identified. As there is currently no national standardised training programme in the UK for perineal assessment and management, including evaluation of clinical practice, the quality and impact of training on uptake of evidence based practice is unknown.

Perineal pain is a commonly reported symptom of maternal morbidity and is highly associated with perineal trauma [2,27]. Recommendations for appropriate pain relief and promotion of advice on perineal hygiene are provided in the NICE guideline for routine postnatal care [28] which highlighted a lack of appropriate information for women on this aspect of their post-birth recovery. Although a commonly experienced symptom, it was apparent that in many cases midwives did not have access to provide women with information on how to manage recovery of their perineal trauma post-birth. The most recent triennial report into maternal deaths in the UK from the Centre for Maternal and Child Enquiries (CMACE) found that, although very rare in the UK, sepsis was the leading cause of direct maternal deaths during 2006 – 2008 [29]. CMACE recommended that all antenatal and postnatal women should be offered advice on the signs and symptoms of life threatening conditions, including sepsis. The team also recommended that this information should include the importance of good hand and perineal hygiene and need to seek immediate medical care if feeling unwell.

There are several potential limitations to this study. Surveys are useful to collate data from a large sample size at one point in time, but do not provide an opportunity to address the context of care and are subject to potential responder bias [30]. Despite the low overall response rate which was a consequence of up to date details on RCM members not being collated by the College and thus not available to the authors, a high number of responders met our inclusion criteria and were representative of the current midwifery workforce. The larger number of midwives qualified for 20 years or longer reflects the age profile of the current UK midwifery workforce, with around 4% of midwives working outside of the NHS [31]. The numbers of midwives working in different models of care was also reflective of current maternity service organisation [32], although it is less clear if the geographic location of care was representative of current service availability.

## Conclusions

This survey has identified considerable gaps with implementation of evidence by midwives in practice to support the management of birth related perineal trauma. Historically, it is acknowledged that the provision of training in assessment and management of perineal trauma has been neglected. Changes in perineal management interventions in the UK, such as reduction in routine use of episiotomy and the low number of repairs performed by midwives as reported in our survey, may now be contributing to a low level of confidence among midwives to assess trauma and perform perineal repair, particularly among those who qualified more recently. The low number of repairs being undertaken by midwives is also likely to influence midwifery views of their skills and competencies, with some indication from midwife comments that a lack of opportunity was preventing them from developing these. Further exploration is required of midwives’ use of knowledge to inform their clinical decisions, given the relatively high proportion who would not suture second degree tears despite national recommendation. The need to address these issues through provision of nationally agreed standards for training is essential if women’s short and longer-term health is to benefit from evidence based interventions. Survey findings have informed the content and delivery of the PEARL study national clinical quality improvement study, results from which will be published separately.

### Details of ethics approval

Ethics approval was obtained from the Thames Valley Ethics Committee (07/MRE12/12).

## Competing interests

The members of the PEARLS study group declare they have no financial or non-financial competing interests in the research presented.

## Author’s contributions

DB and CK conceived the original study, and DB, CK, SM, KMI, ST and PT contributed to the study design. All authors edited the manuscript and read and approved the final manuscript. DB is the study guarantor.

## Pre-publication history

The pre-publication history for this paper can be accessed here:

http://www.biomedcentral.com/1471-2393/12/57/prepub
